# Recurrent Primary Spontaneous Pneumothorax in an 18-Year-Old Man: A Case Report and Review of Considerations for Prophylactic Contralateral Pleurodesis

**DOI:** 10.7759/cureus.110257

**Published:** 2026-06-04

**Authors:** Louise Nicolette Mendoza, Wyatt Mayer, Mario Loomis

**Affiliations:** 1 Department of Clinical Anatomy, Sam Houston State University College of Osteopathic Medicine, Conroe, USA

**Keywords:** chest tube, pleurodesis, primary spontaneous pneumothorax, risk factors for pneumothorax, video-assisted thoracoscopic surgery (vats)

## Abstract

Primary spontaneous pneumothorax (PSP) is defined as the accumulation of air in the pleural space originating from within the lung parenchyma without preceding trauma, which can lead to a stable or tension pneumothorax. Clinical suspicion is key for diagnosis, with chest radiography serving as the primary diagnostic tool. Management ranges from needle aspiration to tube thoracostomy with consideration of surgical intervention for recurrent cases. We present an 18-year-old African-American man who experienced three episodes of radiography-confirmed PSP occurring over a seven-month period. This case is notable for its recurrent pattern of PSP, including contralateral involvement after unilateral pleurodesis, in an adolescent who is otherwise healthy. This case underscores the challenges posed by idiopathic PSP and the need to evaluate genetic or structural predispositions, while raising the question of whether prophylactic contralateral pleurodesis should be considered in patients with recurrent unilateral PSP. The rarity of such recurrence in adolescents supports further research and development of standardized evaluation algorithms in this population.

## Introduction

Pneumothorax (PTX) is defined as the presence of air in the pleural space that can lead to partial or complete collapse of the lung [1]. PTX can be categorized into two types: spontaneous and non-spontaneous. Spontaneous PTX occurs without an apparent precipitating event and is divided into primary or secondary [1]. Primary spontaneous PTX (PSP) typically occurs in otherwise healthy, tall, thin, young individuals and is more common among smokers [1-3]. Research from the Danish National Patient Registry for 2009-2014 found that the peak annual incidence of first PSP occurred between ages 16 and 20 [2]. Reported risk factors for recurrence of PTX include male sex, tall and thin body habitus, apical blebs, and smoking status [1-3]. Overall recurrence after a first PSP is relatively common, reported in roughly 20-30% of patients over several years of follow-up [3]. Secondary spontaneous PTX occurs in individuals with underlying lung disease such as chronic obstructive pulmonary disease, asthma, tuberculosis, or cystic fibrosis [1]. Non-spontaneous PTX can occur due to trauma or iatrogenic causes [1].

PTX clinically presents with sudden-onset chest pain and shortness of breath. Diagnosis is confirmed with a chest radiograph. Treatment depends on size, severity, type, and the patient’s clinical condition [1-3]. A clinically stable patient presenting with a small PTX is managed with observation, supplemental oxygen, and follow-up imaging in 24-72 hours [1,2,4]. Large or symptomatic PTX requires intervention with needle aspiration or chest tube insertion to evacuate the air from the pleural space and to allow the lung to re-expand [1,2,4]. After recurrence, surgical intervention is generally recommended. Video-assisted thoracoscopic surgery (VATS) is recommended after a second ipsilateral PTX, first contralateral PTX, persistent air leak after five days, or failure of lung re-expansion [1-4]. VATS involves resection of blebs and pleurodesis. Mechanical pleurodesis is a surgical procedure that induces inflammation to stick the visceral pleura (lung) to the parietal pleura (chest wall). This procedure helps prevent recurrent PTX by minimizing pleural cavity volume [1-4]. Pain control and infection prevention are important aspects of management [1-4]. Major international guidelines, specifically the joint European Respiratory Society (ERS), the European Association for Cardio-Thoracic Surgery (EACTS), and the European Society of Thoracic Surgeons (ESTS) 2023, do not make recommendations on prophylactic contralateral procedures, such as pleurodesis, at the time of ipsilateral surgery for PSP [5]. The task force cited insufficient evidence to support or oppose prophylactic treatment of the contralateral pleural space [5]. We report a case of recurrent PSP in a young male and discuss the potential role of prophylactic contralateral pleurodesis in this setting.

## Case presentation

An 18-year-old African-American man from Southeast Texas with no family history of PTX, connective tissue disease, or cystic lung disease presented with left-sided spontaneous PTX, which recurred after six weeks and was treated with pleurodesis. Four months later, he developed a right-sided spontaneous PTX. In October 2025, he presented to the emergency department (ED) with right-sided chest pain and dyspnea. He was sitting down when the pain began, with symptoms aggravated by deep inspiration. He denied any trauma, cough, fever, or chills. He presented similarly to his prior PTX episodes, consisting of pleuritic chest pain that developed at rest and difficulty breathing.

The first episode occurred in April 2025, when the patient presented to the ED with complaints of midsternal moderate chest pain that worsened when he took a deep breath. He stated that the pain began when he woke that morning and denied any chest pressure, falls, injuries, fever, chills, or cough. During this episode, the patient was diagnosed with a 20% left PTX, and a left-sided 14 French chest tube was placed. The patient’s symptoms improved, and a repeat chest radiograph showed proper chest tube placement and resolution of PTX. The patient was then transferred to a higher level of care for management of the chest tube. The tube was kept in until there was no recurrence of PTX with the chest tube off suction.

The second episode occurred six weeks later in June 2025, when the patient presented to the ED with similar symptoms. He was sitting at home when he developed chest pain and shortness of breath. He denied any trauma, smoking, or diaphoresis. A 14 French left-sided chest tube was placed with improvement of symptoms, and the chest tube was managed as before. The general surgery team was then consulted, and a left VATS with wedge resection, mechanical pleurodesis, fiberoptic bronchoscopy, and intercostal nerve block was recommended and performed. His postoperative course included hospitalization for management of pain and chest tube drainage. The patient was stable at discharge.

Physical examination findings

Physical examination revealed a healthy-appearing young man in no acute distress, sitting upright and taking shallow breaths, with breath sounds present bilaterally. Vital signs on presentation were a respiratory rate of 17/min, oxygen saturation of 100% on room air, blood pressure of 126/71 mmHg, and heart rate of 63/min. Other findings included a height of 180 cm, a weight of 140 lbs, and a BMI of 19.5. There were no abnormal findings or trauma noted.

Diagnostic studies

**Figure 1:**
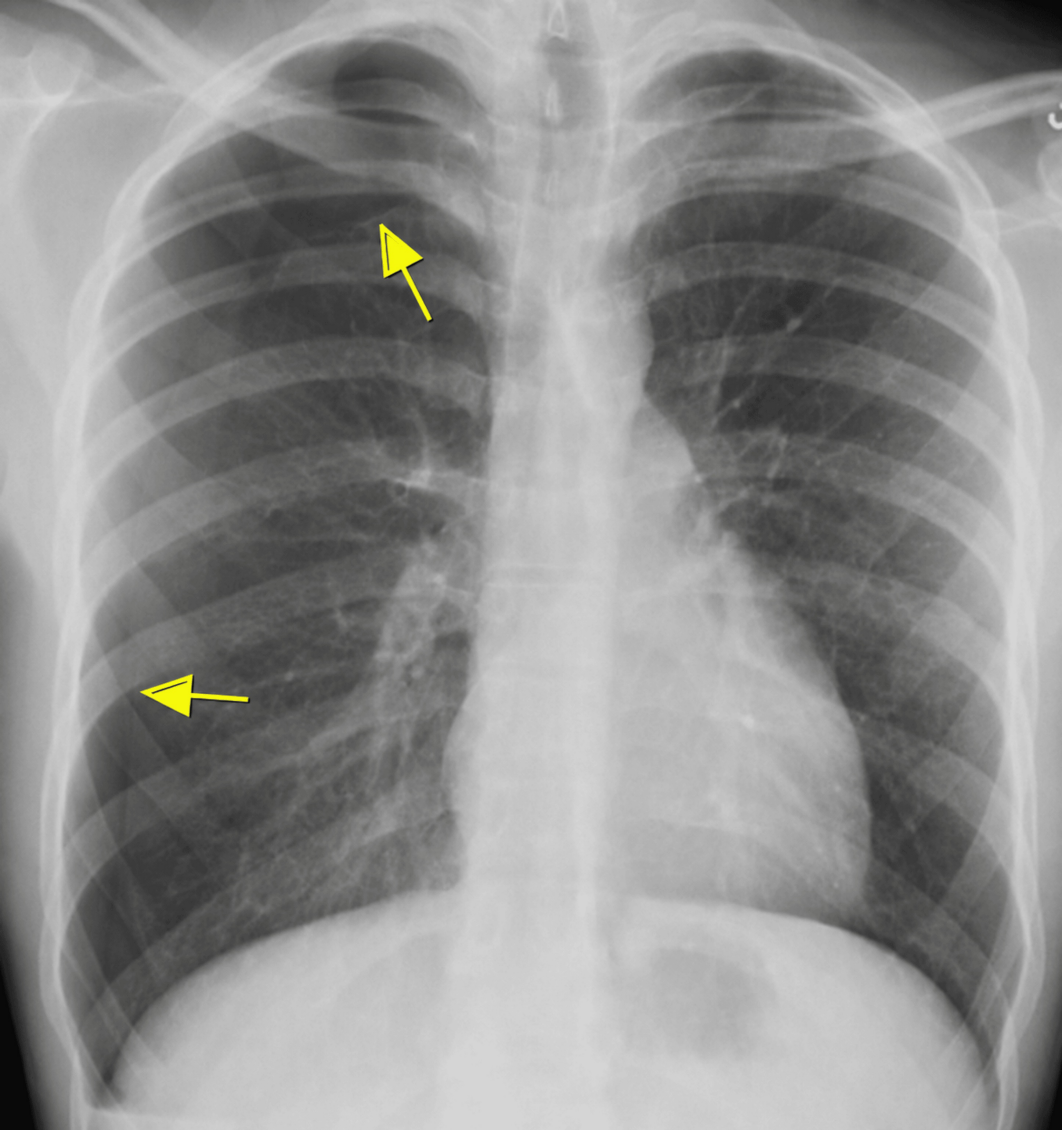
Chest radiograph on presentation showing moderate right-sided PTX (arrows) PTX: pneumothorax

**Figure 2:**
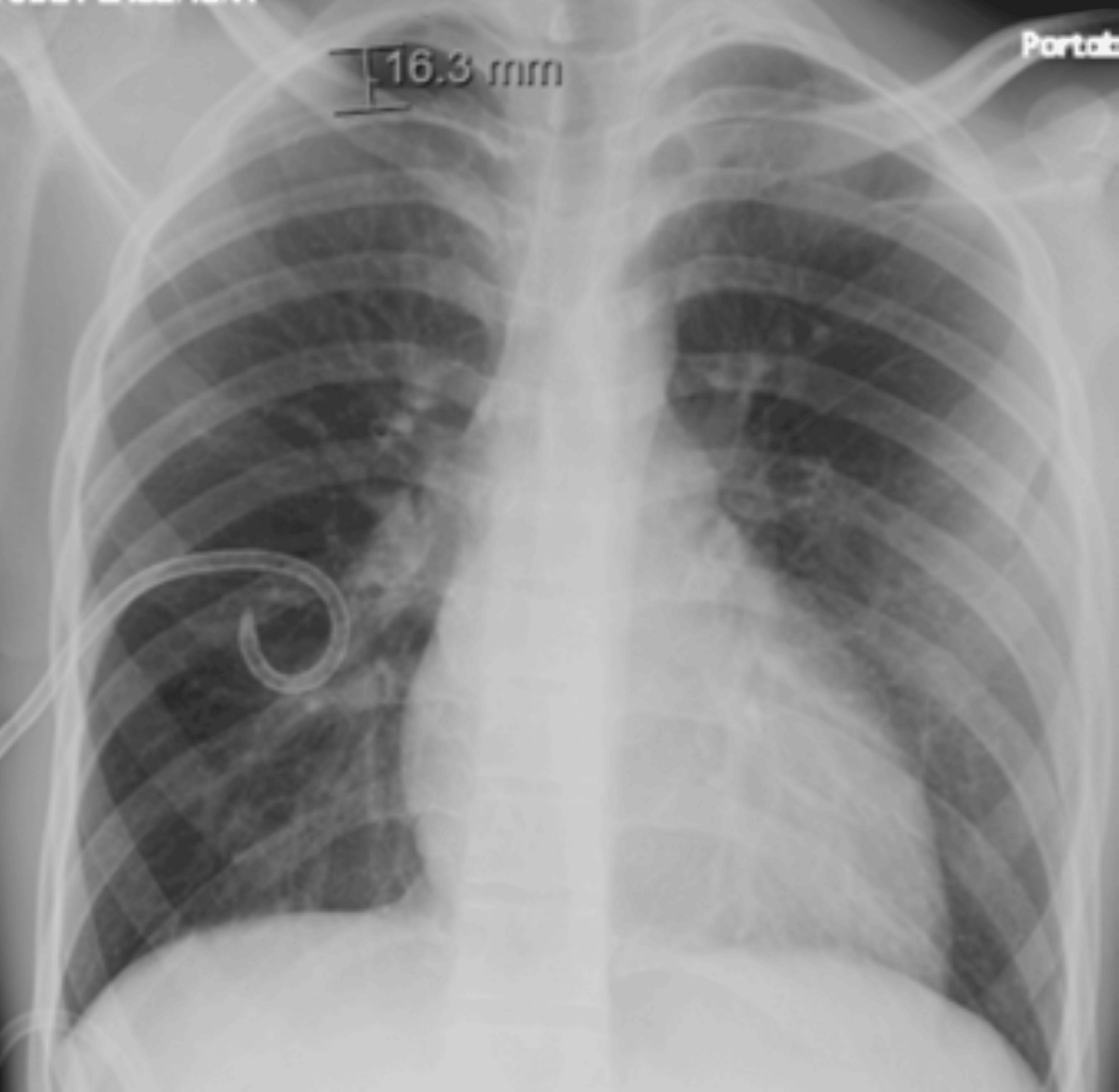
Chest radiograph following pigtail catheter placement confirming catheter position, lung re-expansion, and resolution of the PTX Note the reduction in PTX compared to Figure 1. PTX: pneumothorax

Chest radiography demonstrated a moderate right-sided PTX (Figure 1). A pigtail catheter was placed on the right side, and a repeat chest radiograph was taken to confirm placement, re-expansion of the lung, and resolution of the PTX (Figure 2). Morphine and Zofran were given after the procedure for pain management and antiemetic therapy.

## Discussion

The presentation of a new PSP on the contralateral side of a recurrent PTX is a significant clinical event, occurring in approximately 14-15% of cases. While often stemming from the same underlying pathophysiology as the initial event, namely, the rupture of undetected, bilateral subpleural blebs or bullae, contralateral recurrence is a critical indicator that should prompt investigations for structural or genetic predispositions [6-8]. Some genetic or cystic lung diseases associated with recurrent PTX include vascular Ehlers-Danlos syndrome, Birt-Hogg-Dubé syndrome, and pulmonary Langerhans cell histiocytosis [2,6,7]. The subsequent contralateral involvement in this adolescent raises important questions about strategies to prevent contralateral occurrence, including prophylactic pleurodesis. Observational data suggest that contralateral events occur in more than half of PSP populations; more than half of patients have contralateral blebs or bullae on imaging, and approximately one quarter of those with contralateral blebs may later develop a contralateral PTX [7,8]. Factors associated with recurrence include demographic and lifestyle characteristics such as younger age, greater height, male sex, and a history of smoking [1,2,3,8]. In this context, an otherwise healthy-appearing adolescent who met criteria for pleurodesis with multiple left PTX over a short interval represents a case in which prophylactic treatment on the other side or further investigation for contralateral blebs may need to be considered.

Several surgical series have explored pre-emptive or bilateral VATS approaches. A prospective study found that patients who underwent bilateral VATS with treatment of asymptomatic contralateral blebs experienced no recurrence on either lung during approximately 17 months of follow-up, while unilateral VATS in patients with contralateral blebs was associated with contralateral PTX in 17.14% over a similar period [9]. Other cohorts report that contralateral PSP occurs in approximately 10-18% of patients overall [10]. However, the rate may be as high as one-third in patients with documented contralateral bullae [10]. Because of this increased risk, some authors have proposed single-stage bilateral surgery in young, underweight, or recurrent PSP patients who have contralateral bullae on high-resolution computed tomography [9]. However, pleurodesis and wedge resection can come with many complications such as significant pain, infection, breathing difficulties, or persistent air leak. Air leaks are amongst the most common complications of elective wedge resection of the lung [11]. Air leak rates are reported to range from 20% to 33% after an elective pulmonary resection [11]. Most of these air leaks seal on their own, often within five days of surgery, and are managed with observation and tube thoracostomy [9]. These findings support the concept that both further investigation of the presence of contralateral blebs and potential prophylactic treatment of contralateral pathology can reduce contralateral recurrence in carefully selected high-risk patients, particularly young, tall, lean male smokers [8,10]. Contralateral PTX occurred in 18.1% of patients aged ≤19 years, compared with 4.6% among patients aged 20-29 years [12]. In adolescent and young adult populations, systematic reviews note the lack of consensus regarding prophylactic management of the contralateral side and emphasize individualized decision-making based on recurrence risk and patient factors [3-5].

However, major international guidelines currently stop short of recommending prophylactic contralateral pleurodesis at the time of ipsilateral surgery. The 2023 ERS/EACTS/ESTS guideline emphasizes evidence gaps regarding recurrence-prevention strategies and specifically notes insufficient data to support or refute additional interventions such as contralateral pleurodesis or routine treatment of contralateral blebs during the index operation [5]. Large contemporary datasets also show that while contralateral bullae significantly increase the relative risk of future contralateral PSP, the absolute annual event rate is modest (around 4% per year and decreasing over time), leading some investigators to recommend a conservative approach to unruptured contralateral lesions outside selected high-risk scenarios [3,5,10]. Thus, any prophylactic contralateral procedure must balance potential benefit in reducing contralateral events against operative risk, longer anesthesia time, postoperative pain, and the possibility of overtreatment in patients who might never have a PTX on that side [5].

Our case lies at the intersection of these competing considerations. Although the patient’s prior primary spontaneous pneumothoraces were clinically stable, his body habitus confers an increased risk of underlying structural lung abnormalities, such as apical blebs or bullae. Additionally, his residence in a relatively medically underserved region may delay recognition and timely decompression, thereby increasing the risk that future episodes might progress to a tension PTX. Evaluation of etiology and long-term management strategies for recurrent PTX in adolescents are not standardized, and many patients undergo repeated chest tube placement for each acute episode. This case describes an adolescent male with recurrent PSP over seven months, including early contralateral PSP after ipsilateral mechanical pleurodesis, a pattern associated with an increased risk of contralateral disease [3,4,10]. While our case highlights the question of whether prophylactic treatment of the uninvolved side in primary PTX is warranted, it also highlights the importance of counseling patients about the need for follow-up assessment after recurrence to evaluate for underlying genetic or cystic lung disease and to discuss preventative options [6,7].

This report is limited by the absence of longitudinal outcome data and lack of follow-up after right chest tube treatment. The report is also limited by the lack of prior evidence of contralateral blebs in this patient and by the patient's treatment in a rural setting with limited imaging capabilities. There is no prior evidence that the patient underwent computed tomography imaging. Nonetheless, it reinforces the value of structured follow-up and the need for standardized diagnostic pathways. Further reports and long-term follow-up studies would help clarify the mechanism of recurrence and guide care for populations predisposed to recurrent PTX, such as those with documented blebs and a prior history of PSP on either side.

## Conclusions

This case underscores that PSP in adolescents, while rare, can recur ipsilaterally or occur contralaterally. Existing guidelines do not recommend routine prophylactic contralateral pleurodesis, reflecting limited and heterogeneous evidence, yet emerging data suggest that young patients with recurrent PSP and contralateral bullae have a substantially higher risk of contralateral PTX and may benefit from pre-emptive contralateral treatment. Our case, in particular, highlights the need for further studies to guide prophylactic interventions and standardize evaluation in young patients with recurrent PSP.
